# Chemical Diversity of *Artemisia rutifolia* Essential Oil, Antimicrobial and Antiradical Activity

**DOI:** 10.3390/plants12061289

**Published:** 2023-03-13

**Authors:** Elena P. Dylenova, Svetlana V. Zhigzhitzhapova, Elena A. Emelyanova, Zhargal A. Tykheev, Daba G. Chimitov, Danaya B. Goncharova, Vasiliy V. Taraskin

**Affiliations:** 1 Baikal Institute of Nature Management, Siberian Branch, Russian Academy of Sciences, 670047 Ulan-Ude, Russia; edylenova@mail.ru (E.P.D.); zhig2@yandex.ru (S.V.Z.); emelianowa.elena2312@mail.ru (E.A.E.); danaydomi5@gmail.com (D.B.G.); vvtaraskin@binm.ru (V.V.T.); 2 Institute of General and Experimental Biology, Siberian Branch, Russian Academy of Sciences, 670047 Ulan-Ude, Russia; dabac@mail.ru

**Keywords:** *Artemisia rutifolia*, essential oil, chemical composition, monoterpenes, sesquiterpenes, PCA-analysis, chemotypes, antibacterial activity, antiradical activity

## Abstract

This paper presents the results of the study of the composition of the essential oil (EO) of *Artemisia rutifolia* by the GC/MS method as well as its antimicrobial and antiradical activities. According to the PCA-analysis, these EOs can be conditionally divided into “Tajik” and “Buryat-Mongol” chemotypes. The first chemotype is characterized by the prevalence of *α*- and *β*-thujone, and the second chemotype by the prevalence of 4-phenyl-2-butanone, camphor. The greatest antimicrobial activity of *A. rutifolia* EO was observed against Gram-positive bacteria and fungi. The EO showed high antiradical activity with an IC_50_ value of 17.55 μL/mL. The presented first data on the composition and activity of the EO of *A. rutifolia* of the Russian flora indicate the prospects of the species as a raw material for the pharmaceutical and cosmetic industry.

## 1. Introduction

Essential oils are a mixture of volatile flavor substances belonging to different classes of organic compounds (terpenes, their oxygenated derivatives, aromatic and aliphatic compounds). These compounds can pass through biological membranes to exert antioxidant, antimicrobial, antifungal, anti-inflammatory, antiviral, and other effects [1], making EOs widely used in the pharmaceutical and cosmetic industries and increasing the demand for new natural sources of EOs.

Plants of the *Artemisia* L. genus, which grow abundantly in arid and semi-arid regions of Asia, can serve as a reliable natural source of EOs. A promising species is *Artemisia rutifolia* Steph. ex Spreng. (family Asteraceae Bercht. Et J. Presl., section *Absinthium* (Mill.) D.-C.), which is a semi-shrub, up to 80 cm tall with strongly branched, woody perennial stems covered with brownish grey, cracked bark [2]. It grows in Afghanistan, Kazakhstan, Kyrgyzstan, Mongolia, Nepal, Pakistan, Russia (Western and Eastern Siberia), Tajikistan, and Western Asia [3], in mountain steppes, rocky slopes, and screes [4]. On the territory of Baikal Siberia, *A. rutifolia* is a relict species [5], the life expectancy of which can reach 80–90 years [6]. In Kyrgyzstan folk medicine, fresh leaves have been used for toothache, and a decoction for sore throat, heart, and stomach diseases [7]. The therapeutic value of the species exhibited is due to the variety of biologically active substances it contains.

The isolation of sesquiterpene lactones (guyanolides, germacranolides, and costic acid derivatives) from the aerial part of *A. rutifolia* has been reported [8,9,10]. Another study reported that methanol, chloroform, and hexane extracts of *A. rutifolia* leaves contained polyphenolic compounds (organic acids, myricetin, and quercetin) and also exhibited antimicrobial and antioxidant activities [11]. The following terpenes were isolated by gas chromatography and identified by their IR spectra from the EO of *Artemisia rutifolia*: 1,8-cineole, *α*-, *β*-thujones, (+)-camphor, (−)-*α*-terpineol, and (−)-terpinen-4-ol [12]. However, studies of the essential oil composition of *A. rutifolia* were generally incomplete and related to plants growing in scattered populations from Tajikistan [13] and Mongolia [12,14,15,16].

This article is the first to investigate the chemical composition of the EO of *A. rutifolia*, growing in Buryatia (Russia), its antimicrobial and antiradical activities, and to conduct comparative chemometric analysis.

## 2. Results and Discussion

### 2.1. EOs Component Composition

**Figure 1:**
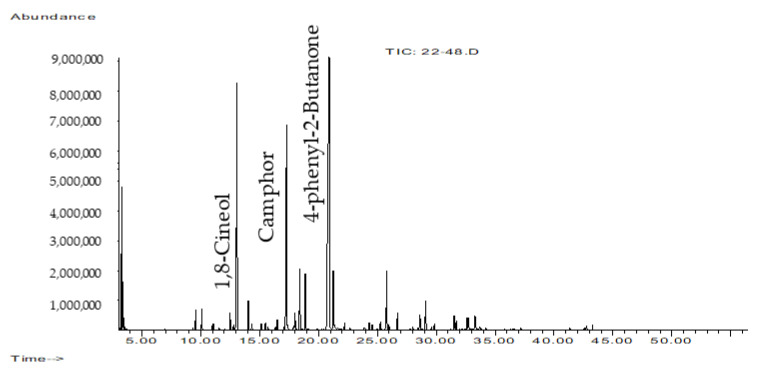
GC-MS total ion chromatogram of *A. rutifolia* EOs.

The yield of EOs from the aerial part of *A. rutifolia* growing in Buryatia (Russia) was 1.82% (*v*/*w*) of dry weight. The chemical composition of the obtained EOs was investigated using the GC-MS technique (Figure 1). Forty components have been identified in the EO of *A. rutifolia*, most of which are represented by mono- and sesquiterpenoids, and are listed in Table 1. The dominant components were: 4-phenyl-2-butanone (34.98%), 1,8-cineol (16.53%), camphor (16.67%), also in significant quantities were found: terpinen-4-ol (3.71%), 4-phenyl-2-butanol (3.58%), α-terpineol (3.51%), α-methyl-benzenepropanol acetate (3.43%), bicyclogermacrene (2.06%), and germacrene D (1.02%). Monoterpenes (51.26%), especially the oxygenated ones (45.28%), made up the largest proportion of all components.

### 2.2. Chemical Diversity of EOs

Comparative analysis of the obtained data and the literature review [13,14,15,16] (Appendix A) showed that the EO of plants growing in Buryatia was similar to the EOs of Mongolian plant populations in the content of the major components, but quite different from the EOs of plants from Tajikistan.

Thus, the dominant components in the EOs of *A. rutifolia* from the Muminobod and Yovon regions of Tajikistan were *α*-thujone (20.9–36.6%), *β*-thujone (36.1–47.3%), 1,8-cineol (3.2–11.7%), myrcene (0.3–2.8%), *p*-cymol (0.9–1.8%), *cis*-piperitone epoxide (0.9–2.0%), and germacrene D (1.8–2.8%). More than 90% of all components were monoterpenoids, mainly oxygenated (85.5–92.4%).

In contrast, the dominating components of the EOs of plants from the Mongolian populations were: 4-phenyl-2-butanone (33.1%), carvacrol methyl ether (29.58%), camphor (2.13–22.4%), 1,8-cineol (4.63–25.13%), 4-phenyl-2-butanol (3.4%), geraniol (2.91%), *p*-cymol (1.1–1.41%), *α*-terpineol (1–1.64%), *α*-thujone (0.7–3.38%), *β*-thujone (1.10–3.2%), and terpinen-4-ol (0.54–1.1%). Monoterpenoids (58.26–93.37%) also dominated among all of the other components.

Samples from Mongolia and Buryatia (compared to those from Tajikistan) were characterized by a sufficiently high content of camphor, which is used in creams, ointments, and lotions to relieve pain, irritation, itching, and has antifungal and antibacterial properties [17].

**Figure 2:**
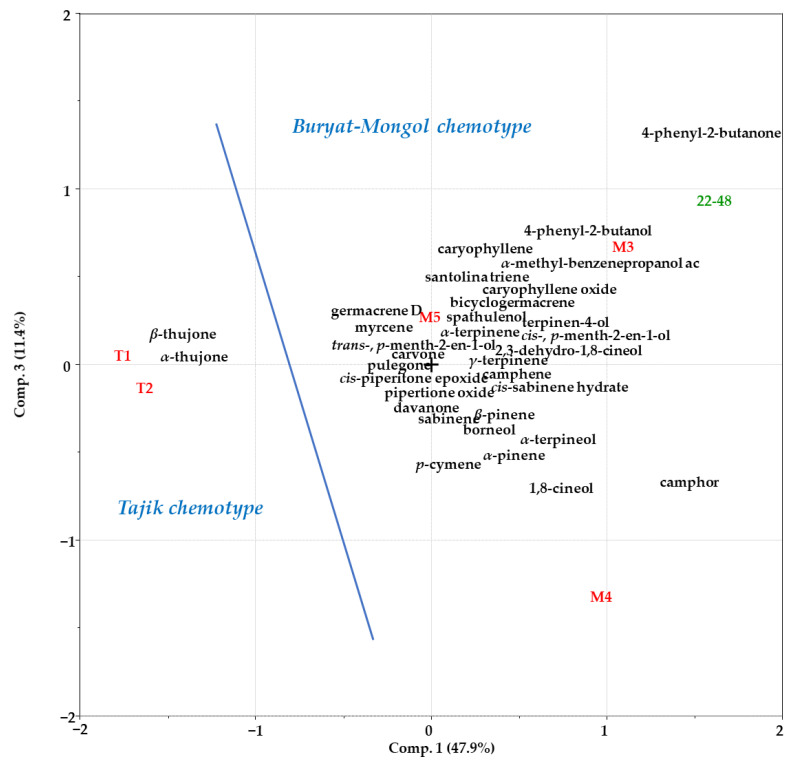
PCA biplot (principal component 1–principal component 3) for the data on the composition of *A. rutifolia* EOs.

Using PCA to compare our own and the literature data on the content of the major components of *A. rutifolia* EO, it was shown that these EOs can currently be conditionally divided into the “Tajik” and “Buryat–Mongol” chemotypes (Figure 2).

**Figure 3:**
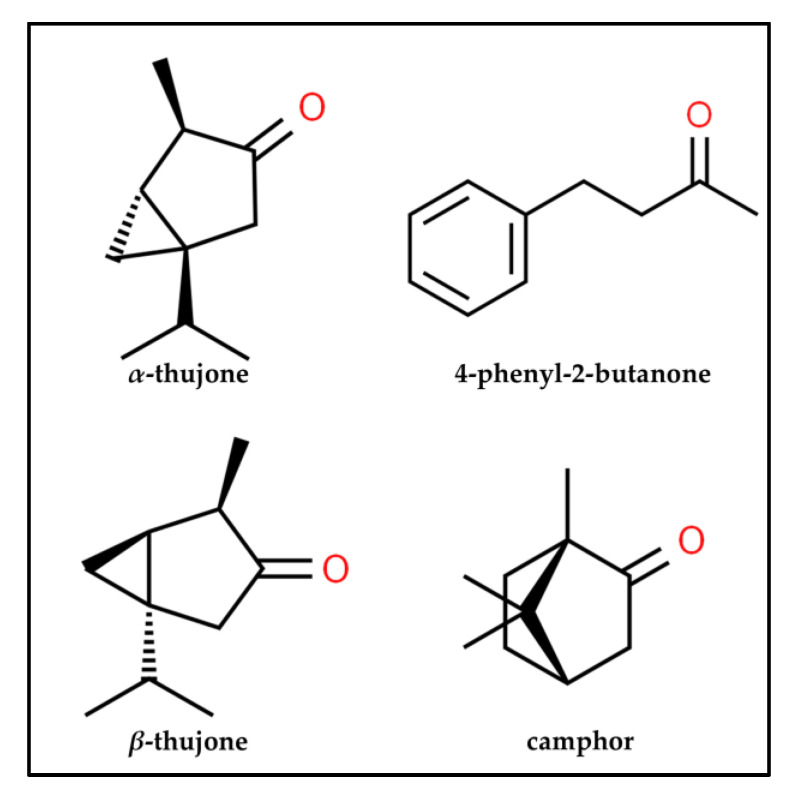
Chemical structure of the major compounds forming different chemotypes of *A. rutifolia*.

The “Tajik” EOs were characterized by the prevalence of *α*- and *β*-thujone, while the “Buryat-Mongolian” chemotype was characterized by a high content of 4-phenyl-2-butanone, camphor (Figure 3).

For example, in the EOs of *A. rutifolia* from the flora of Tajikistan [13] the content of *α*- and *β*-thujone was rather high: *α*-thujone (20.9–36.6%) and *β*-thujone (36.1–47.3%), whereas in the EOs of the plants from Mongolian populations [14,15,16] they were found in smaller amounts: *α*-thujone (0.70–3.38%), *β*-thujone (1.10–3.20%). However, they were not found in the plants of the Buryat flora.

It should be noted that studies of isomeric thujones (*α*- and *β*-) were previously initiated because wormwood is widely used to flavor alcoholic beverages. The most famous alcoholic beverage, absinthe, is made from *Artemisia absinthium*.

Thujones are known to be the main constituents of the EOs of *A. absinthium* [18]. It has neurotoxicity manifested by hyperactivity, tremors, and tonic convulsions [19]. The effects of thujone on the human body are related to the inhibition of GABAA receptors, leading to dose-dependent excitation and convulsions, with (−)-*α*-thujone having a greater ability to induce convulsions than the (+)-*β*-isomer; it is more likely that the convulsive effect of thujone acts on a specific receptor system [20]. For this reason, isomeric thujones were long thought to be responsible for the manifestation of the so-called “wormwood epilepsy”.

Modern studies show that it is the additional components (apart from the main one—ethyl alcohol) of industrially produced absinthe that do not seem to have any harmful effects on health, leaving aside the effects of ethanol on the body. Absinthe has an exceptionally high alcohol content (>50% vol.). This can lead to serious health and social problems, but it is not unique to this drink. So-called “absinthism” cannot be clearly distinguished from chronic alcoholism [21].

In general, thujones (monoterpene ketones) are natural constituents of the EOs of plants of the genus *Artemisia* (*A. absinthium, A. campestris, A. alba, A. incana, A. pontica, A. santolinifolia, A. santonicum, A. spicigera, A. vulgaris*), Salvia (*S. fruticosa, S. lavandulifolia, S. officinalis, S. sclarea, S. triloba*), Thuja (*T. occidentalis, T. orientalis*), etc. [19]. However, the assessment of thujone toxicity remains poorly studied, the most important aspects of which are the relationships between dose, concentration, and effect in humans.

The content of thujones in the EO of *A. absinthium* can vary within a wide range. On this basis, thujone and sabinyl acetate EOs of *A. absinthium* were distinguished [22]. Thujone-containing and thujone-free forms are also characteristic of other wormwood species (e.g., *A. campestris* [23], *A. molinieri* [24]).

On the other hand, the discovery of thujone-free forms of *A. rutifolia* growing in Buryatia is important for the creation of safer medicines, cosmetics, food supplements, and therapeutic foods based on them. In addition, it allows us to understand the influence of environmental conditions on thujone biosynthesis. The currently available amount of information on the composition of EOs of *A. rutifolia* does not allow us to draw detailed conclusions, but we note that the formation of chemotypes occurs under the influence of a long-term and relatively uniform action of certain climatic conditions. In the course of evolution, changes in the composition of enzymes occur by replacing one or more amino acids. If the modified enzyme produces a useful product for the plant, these changes are fixed in the genes [25].

At the biochemical level, mechanisms are formed to synthesize a specific set of enzymes that contribute to the production of EO components of one or another chemotype. The biosynthesis of thujones has been studied in detail for only a few species. It is known that the first monoterpene in this transformation chain is sabinene, whose formation is catalyzed by the enzyme sabinene synthase. Furthermore, isomeric thujones are formed from isomeric sabinols, probably also from (+)-sabinone [26].

The territories of Tajikistan, Mongolia, and Buryatia (Russia), where *A. rutifolia* grows, belong to the arid zone of Asia. The territories of Buryatia and Mongolia belong to the eastern (and Tajikistan—to the western) longitudinal sector of the arid continental zone of Asia, where the most arid territory is Mongolia. The *eastern boundary* of the extremely arid deserts of southern Mongolia and northern China, which have no analogues in Eurasia, passes here at about 105 degrees east latitude. The harsh natural conditions are particularly pronounced in areas of high aridity in the continental winter climate zone. At the same time, the area where the plants were collected in Tajikistan is on the border of the *western sector*: the interaction of various circulation processes leads to a strong variability in the moisture regimes (there is almost no precipitation in summer). However, the climate of a particular area was influenced by meso- and microclimatic factors in addition to the macroclimatic factors.

The area of plant collection in Mongolia is located in the Great Lakes basin, the mesoclimate of which is close to the semi-arid climate of Buryatia [27]. Thus, these places where the raw materials were collected can be ranked as follows (in the order of increasing the aridity of growing conditions of plants in summer) Buryatia → Mongolia → Tajikistan.

The increasing aridity of climatic conditions will likely lead to the biosynthesis of thujones. In addition, other sabinene derivatives, *trans*- and *cis*-sabinene hydrates, have been found in small amounts in the EOs of *A. rutifolia* growing in the territories of Buryatia and Mongolia; in Mongolian plants, sabinyl acetate was found. These compounds probably block thujone biosynthesis.

### 2.3. Antimicrobial Activity

The antimicrobial activity of *A. rutifolia* EO was experimentally determined using the disc diffusion method against Gram-positive bacteria (*Streptococcus pyogenes, Staphylococcus aureus, Bacillus cereus*), Gram-negative bacteria (*Escherichia coli, Pseudomonas aeruginosa, Salmonella enterica*), and fungi (*Aspergillus niger*, *Candida albicans*).

The antimicrobial activity of the samples was evaluated by the diameter of the growth inhibition zones of the test strains (mm). Each sample was tested in three replicates. The test results of the antimicrobial activity of the samples are shown in Table 2.

The results indicate the greatest antimicrobial activity of *A. rutifolia* EO against Gram-positive bacteria (*Streptococcus pyogenes*, *Staphylococcus aureus*, *Bacillus cereus*) and fungi (*Aspergillus niger*, *Candida albicans*), with pronounced activity against Aspergillus niger.

To a lesser extent, the growth inhibition of Gram-negative bacteria (*Salmonella enterica subsp. enterica*, *Escherichia coli*) was observed. *Pseudomonas aeruginosa* proved to be the most resistant to the EO: no growth inhibition was observed.

The greatest antimicrobial activity of *A. rutifolia* EO from Buryatia was observed against the Gram-positive bacteria and fungi, which is consistent with the literature data. For example, the minimum inhibitory activity (MIC) and minimum bactericidal concentration (MBC) of the EOs of *A. rutifolia* from Tajikistan were previously determined to be 10 mg/mL against *E. coli* ATCC 25922, and 5 mg/mL against MRSA NCTC 10442 [28].

EOs of *A. rutifolia* from Mongolia at a concentration of 150 mg/mL (or 3 μg/disc) inhibited the growth of *S. enterica* by 9.3 ± 0.76 mm, *B. subtillus* by 10.3 ± 0.58 mm, and *S. aureus* by 9.6 ± 1.5 mm, thus showed moderate antimicrobial activity [16]. The target for the antimicrobial action of the EO is probably the bacterial cell wall, which is known to be fundamentally different in structure in Gram-positive and Gram-negative bacteria. The cell wall of Gram-negative bacteria contains a strong lipid layer on its surface, with which the EOs lose their antimicrobial activity [29]. Therefore, the *A. rutifolia* EO is recommended for use as an antimicrobial agent against Gram-positive bacteria and fungi.

### 2.4. Antiradical Activity

**Figure 4:**
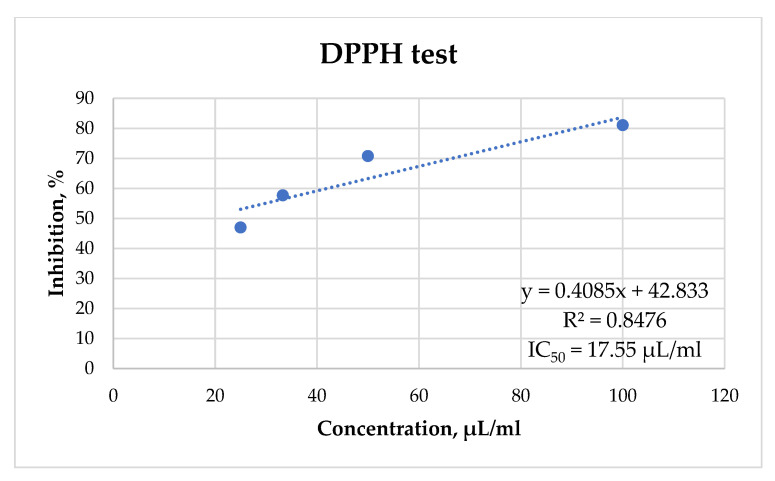
DPPH test of the antiradical activity of *A. rutifolia* EO.

In order to evaluate the possible antiradical potential of the EO of *A. rutifolia*, the DPPH test (2,2-diphenyl-1-picrylhydrazyl radical inhibition) was applied. To determine the antiradical properties of the EO, a kinetic curve was constructed using the IС_50_ value (Figure 4).

According to the results of the test, it was found that the EO has high antiradical activity as the IC_50_ value was 17.55 μL/mL.

It is considered that the antioxidant potential of EOs is exhibited mainly due to the presence of oxygenated monoterpenes (especially of phenolic structure), while sesquiterpene hydrocarbons and their oxygenated derivatives have very low antioxidant activity [30]. The EOs from Tajikistan had a better antiradical potential (IC_50_ = 7.91 mg/mL) [28] compared to those from Buryatia (IC_50_ = 17.55 μL/mL) and the content of oxygenated monoterpenes was higher in the EOs of *A. rutifolia* from Tajikistan.

Previously, it has been shown that EOs exhibit much greater activity than their individual components, which may be due to the high percentage of major components, and synergism between the various components of the EO including minor ones [31]. For example, wormwood EOs, whose main components are camphor and 1,8-cineol, always show antiradical activity, while camphor and 1,8-cineol individually do not [32].

It has also been shown that cineol enrichment of the secondary oil fractions of bay laurel and the cube residue of rockrose enhances their antioxidant properties [1]. In the case of *A. rutifolia*, we believe that the EO of the plants from Buryatia has a higher antiradical activity due to the synergistic effect.

## 3. Materials and Methods

### 3.1. Plant Material Collection and EO Production

The aerial part of *A. rutifolia*, collected in 2022 in the Selenginsky District (Buryatia, Russia) during the vegetation period, was used as the object of study. The voucher specimens were identified by Dr. Oleg A. Anenkhonov and deposited at the Herbarium of Institute of General and Experimental Biology SB RAS (UUH 019695, 019696). Data on the sampling locations and EO yield are presented in Table 3 (compared to data from other studies).

EOs were obtained by hydrodistillation from air-dry raw materials (aboveground part of plants, for 3 h) in the year of raw material collection, according to OFS.1.5.3.0010.15 “Determination of essential oil content in medicinal plant raw materials and herbal drugs” with a modified Clevenger apparatus.

### 3.2. Gas Chromatography-Mass Spectrometry (GC-MS) Analysis and Principal Component Analysis (PCA)

The component composition of the EOs was determined by gas chromatography-mass spectrometry (GC-MS) using an Agilent 6890 gas chromatograph (Agilent Technologies, USA) with an HP 5973N mass-selective detector (Hewlett-Packard, Palo Alto, CA, USA) and an HP-5MS capillary column (30 m × 0.25 mm × 0.2 µm; Hewlett-Packard), as previously described in [33].

The principal component analysis (PCA) method was applied to the contents of the EO components (Sirius software package ver. 6.0, Pattern Recognition Systems, a/s, Norway).

### 3.3. Antiradical Activity

The antiradical activity of the EOs was determined by the DPPH test (using a stable radical, 2,2-diphenyl-1-picrylhydrazyl). Briefly, a DPPH solution (0.006% in 95% ethanol) was added to the EO of *A. rutifolia* (25–1000 μL/mL in ethyl alcohol) and incubated for 30 min in the dark at room temperature. The antiradical activity was then determined spectrophotometrically on a ClarioStar Plus multimode plate reader at 517 nm.

The antiradical activity (in % inhibition) was calculated using the formula:

% inhibition of DPPH radicals = [(A_0_ − A_1_)/A_0_] × 100,

where A_0_ is the absorbance of the control sample, A_1_ is the absorbance of the test sample.

The IC_50_ index was determined using regression analysis.

### 3.4. Antimicrobial Activity

The antimicrobial activity of the test samples was determined by the technique of diffusion in dense nutrient media. The inoculum was prepared by the direct suspension of the daily culture colonies of each test strain in a sterile isotonic solution to a density of 0.5 according to the McFarland turbidity standard, which approximately corresponds to a load of 1–2 × 10^8^ CFU/mL. The resulting microbial suspension was applied evenly to the entire surface of the nutrient medium (agar) in three directions using a sterile cotton swab.

Mueller–Hinton agar was used as a nutrient medium for microorganisms with normal nutrient requirements, and Mueller–Hinton agar with the addition of 5% defibrinated blood was used for bacteria with complex nutrient requirements (*Streptococcus pyogenes*). After applying the microbial suspension, sterile paper discs were placed on the agar surface and 10 µL of the test samples was applied (one sample per disc). Factory paper discs with antimicrobial additives (norfloxacin for Gram-positive bacteria, ceftazidime for Gram-negative bacteria, fluconazole for fungi) were used as the positive controls.

Cultures were incubated at 37 °C (22 °C for molds and yeasts). The results were recorded after 24 h of incubation for bacteria and 48 h for mold and yeast. To determine the antimicrobial activity of the samples tested, the diameters of the microbial growth suppression zones around the disks were evaluated. Growth inhibition zones were measured to the nearest millimeter.

## 4. Conclusions

Thus, for the first time, the chemical composition and primary biological activities of the EOs of *A. rutifolia* collected in Buryatia were studied. The greatest antimicrobial activity of the EOs was noted with the Gram-positive bacteria (*Streptococcus pyogenes*, *Staphylococcus aureus*, *Bacillus cereus*) and fungi (*Aspergillus niger*, *Candida albicans*). In addition, it showed antiradical activity, and the IC_50_ index was 17.55 μL/mL. The obtained preliminary results of the antimicrobial and antiradical activities allow us to consider that *A. rutifolia* is a promising raw material for the pharmaceutical and cosmetic industries; however, it is necessary to carry out further studies.

The variability of plants growing within the natural habitat greatly affects the composition of essential oils. Despite the variability in the composition, the volatile substances of plants that form essential oils are the most important chemical markers that are used to solve the issues of chemosystematic or the taxonomic assignments of plants. The analysis of our own and the literature data showed that the EOs of *A. rutifolia* can be conditionally divided into “Tajik” and “Buryat-Mongol” chemotypes. The first chemotype is characterized by the prevalence of α- and *β*-thujone, and the second by the high content of 4-phenyl-2-butanone and camphor. The composition is highly variable and greatly depends on the geographical confinement.

## Figures and Tables

**Table 1:** Chemical composition of EOs extracted from the aerial parts of *A. rutifolia* from different countries.

No.	RI *	Rt	Component	Peak Area (%)	Molecular Formula
1	921	9.17	Tricyclene	0.05	C_10_H_16_
2	926	9.32	*α*-Thujene	0.12	C_10_H_16_
3	932	9.56	*α*-Pinene	0.98	C_10_H_16_
4	947	10.08	Camphene	1.06	C_10_H_16_
5	973	10.95	Sabinene	0.22	C_10_H_16_
6	975	11.06	*β*-Pinene	0.37	C_10_H_16_
7	990	11.55	2,3-dehydro-1,8-Cineol	0.15	C_10_H_16_O
8	1004	12.04	*α*-Phellandrene	0.05	C_10_H_16_
9	1017	12.49	*α*-Terpinene	0.89	C_10_H_16_
10	1024	12.78	*p*-Cymol	0.35	C_10_H_14_
11	1031	13.07	1,8-Cineol	16.53	C_10_H_18_O
12	1058	14.04	γ-Terpinene	1.52	C_10_H_16_
13	1066	14.33	*trans*-Sabinene hydrate	0.37	C_10_H_18_O
14	1088	15.13	Terpinolene	0.37	C_10_H_16_
15	1098	15.48	*cis*-Sabinene hydrate	0.44	C_10_H_18_O
16	1103	15.70	Filifolone	0.19	C_10_H_14_O
17	1121	16.35	*cis*-*p*-Menth-2-en-1-ol	0.26	C_10_H_18_O
18	1126	16.49	Chrysanthenone	0.64	C_10_H_14_O
19	1141	17.06	*trans*-*p*-Menth-2-en-1-ol	0.23	C_10_H_18_O
20	1144	17.29	Camphor	16.67	C_10_H_16_O
21	1162	17.88	Pinocarvone	0.31	C_10_H_14_O
22	1166	17.99	Borneol	1.17	C_10_H_18_O
23	1177	18.41	Terpinen-4-ol	3.71	C_10_H_18_O
24	1191	18.88	α-Terpineol	3.51	C_10_H_18_O
25	1241	20.91	4-phenyl-2-Butanol	3.58	C_10_H_14_O
26	1247	21.25	4-phenyl-2-Butanone	34.95	C_10_H_12_O
27	1287	22.21	Bornyl acetate	0.38	C_12_H_20_O_2_
28	1306	24.30	α-Terpineol formate	0.40	C_11_H_18_O_2_
29	1359	24.56	Eugenol	0.32	C_10_H_12_O_2_
30	1378	25.26	*α*-Copaene	0.48	C_15_H_24_
31	1418	25.79	α-methyl-Benzenepropanol acetate	3.43	C_12_H_16_O_2_
32	1422	26.70	Caryophyllene	0.97	C_15_H_24_
33	1456	27.77	Humulene	0.07	C_15_H_24_
34	1464	28.00	*allo*-Aromadendrene	0.19	C_15_H_24_
35	1477	28.41	Selina-4,11-diene	0.13	C_15_H_24_
36	1484	28.61	Germacrene D	1.02	C_15_H_24_
37	1500	29.09	Bicyclogermacrene	2.06	C_15_H_24_
38	1517	29.59	*γ*-Cadinene	0.16	C_15_H_24_
39	1580	31.52	Spathulenol	1.10	C_15_H_24_O
40	1586	31.70	Caryophyllene oxide	0.60	C_15_H_24_O
Total oxygenated hydrocarbons				41.96	
Total monoterpenes				51.26	
Total sesquiterpenes				6.78	
Total hydrocarbons				41.96	

* RI, retention indices: experimental, for our data (RI, retention index as determined on a HP-5MS column using the homologous series of n-hydrocarbons).

**Table 2:** Antimicrobial activity of essential oil from the aerial part of *Artemisia rutifolia* against Gram-positive, Gram-negative bacteria, and fungi.

Tested Substance	Zone of Inhibition, mm							
	Gram-Positive			Gram-Negative			Fungi	
	*Streptococcus pyogenes*	*Staphylococcus aureus*	*Bacillus* *cereus*	*Pseudomonas aeruginosa*	*Salmonella* *enterica*	*Escherichia* *coli*	*Candida* *albicans*	*Aspergillus* *niger*
Essential oil	14	14	14	0	12	13	11	21
Positive control *	25	28	24	26	27	27	46	37

* Positive control: norfloxacin was for the Gram-positive bacteria; ceftazidime for the Gram-negative bacteria; fluconazole for the fungi.

**Table 3:** Origin of the plant material of *Artemisia rutifolia* and the yield of the essential oils from the aerial part.

Sample Code	Country	Locality	Collection Period	Latitude Longitude	Attitude (m)	Yield of the Essential Oil, *v*/*w* (%)	Source of Data
22–48	Russia	Surroundings of the Novoselenginsk Village, Selenginsky District, Buryatia	14.06.2022	N 51.25556 E 106.431389	549	1.82	Present study
T1	Tajikistan	Khonaobod Village, Muminobod region	02.05.2010	N 38.107547 E 69.966431	1200	0.50	[13]
T2	Tajikistan	Chormaghzak Village, Yovon region	25.07.2010	N 38.417502 E 69.172175	1300	0.80	[13]
M3	Mongolia	Middle Gobi Province	08.09.2007	– *	–	0.20	[15]
M4	Mongolia	–	–	–	–	1.20	[14]
M5	Mongolia	Khrakhiraa Mountain, Uvs aimag	09.2019	–	–	0.96	[16]

* Not specified.

## Data Availability

All data generated or analyzed during this study are included in this published article.

## Appendix A

**Table A1:** The chemical composition of EOs extracted from the aerial parts of *A. rutifolia* from different countries.

No.	Component	Peak Area (%)					
		Our Data	Literature Data				
			T1 [13]	T2 [13]	M3 [15]	M4 [14]	M5 [16]
Monoterpene hydrocarbons							
1	Tricyclene	0.05				0.13	
2	*α*-Thujene	0.12	0.1			0.12	
3	*α*-Pinene	0.98	0.2	tr *	0.4	1.25	0.30
4	Camphene	1.06	0.1		0.8	0.34	0.21
5	Sabinene	0.22	0.3	0.4	0.1	0.27	0.82
6	*β*-Pinene	0.37	0.1	0.1	0.2	0.19	
7	*α*-Phellandrene	0.05	0.5	0.1			
8	*α*-Terpinene	0.89	0.2	0.2			
9	*p*-Cymol	0.35	1.8	0.9	1.1	1.41	
10	γ-Terpinene	1.52	0.5	0.4		0.19	
11	Terpinolene	0.37		0.1		0.1	
12	Santolina triene		0.1				22.38
13	Myrcene		2.8	0.3		0.15	21.84
14	Pseudo-limonene						5.27
15	Limonene					0.18	
16	*E*-*β*-Ocimene		tr				
Total monoterpene hydrocarbons		5.98	2.1	2.5	2.6	4.33	50.82
Oxygenated monoterpenes							
17	*cis*-Sabinene hydrate	0.44			0.4	0.11	
18	2,3-dehydro-1,8-Cineol	0.15			1.2		
19	1,8-Cineol	16.53	3.2	11.7	19.1	25.13	4.63
20	*trans*-Sabinene hydrate	0.37			0.3	0.20	
21	Filifolone	0.19					
22	*cis*-*p*-Menth-2-en-1-ol	0.26			1.5		
23	Chrysanthenone	0.64	0.1	0.8			
24	*trans*-*p*-Menth-2-en-1-ol	0.23	0.9	0.5			
25	Camphor	16.67	0.9	0.2	22.4	21.74	2.13
26	Pinocarvone	0.31	0.1	0.2		0.46	
27	Borneol	1.17	0.2	0.4	0.4	0.65	
28	Terpinen-4-ol	3.71	0.6	1.2	1.1	0.54	0.68
29	α-Terpineol	3.51	0.1	0.3	1	1.64	
30	Bornyl acetate	0.38		tr	0.1		
31	α-Terpineol formate	0.40					
32	Eugenol	0.32					
33	Santolina alcohol		0.4				
34	*trans*-2,3-epoxy Pinane					0.12	
35	Linalool					0.25	
36	*α*-Thujone		20.9	36.6	0.7	3.38	
37	*β*-Thujone		47.3	36.1	3.2	1.10	
38	Chryzanthenone					0.38	
39	*iso*-3-Thujanol		0.3	0.1			
40	*trans*-2-Pinanol					0.55	
41	*trans*-Verbenol				0.3		
42	*p*-Menth-3-en-1-ol		0.1	0.2			
43	Menthone		0.9				
44	Sabina ketone		0.2	0.3			
45	*cis*-Pinocamphone			0.1			
46	Thuj-3-en-10-al		0.2				
47	*p*-Cymen-8-ol			0.1	0.2		
48	*cis*-Piperitol		0.4			0.1	
49	Myrtenol			0.3			
50	γ-Terpineol		tr				
51	*trans*-Piperitol		0.5	0.2			
52	*trans*-Carveol				0.2	0.1	
53	*m*-Cumenol		0.1	0.1			
54	*exo*-2-Рydroxycineol				2.3		
55	*nor*-Davanone			0.1			
56	Pulegone		1	0.3			
57	Carvone		0.9	0.1			
58	Carvacrol methyl ether					29.58	
59	Carvotanacetone		0.1	0.1			
60	Geraniol					2.91	
61	*cis*-Piperitone epoxide		2.0	0.9			
62	*cis*-Chrysanthenyl acetate		0.2	tr			
63	*iso*-3-Thujanol acetone		0.1	0.1			
64	*neoiso*-3-Thujanol acetone			0.1			
65	Sabinylacetate				0.9		
66	*p*-Cymen-7-ol		0.1	tr			
67	Thymol		0.7	0.2			
68	Carvacrol		0.9	0.4	0.1		
69	*Z*-Patchenol			0.2			
70	*cis*-Piperitol acetate		0.1	0.1			
71	Piperitone		0.1	0.1			
72	Pipertione oxide		1.4	tr			
73	*trans*-Carvylacetate				0.2		
74	*α*-Terpenylacetate				0.3	0.1	
75	*Z*-Jasmone		0.1	0.3	tr		
76	Methyleugenol				tr		
77	*E*-Ionone		0.1	tr			
Total oxygenated monoterpenes		45.28	85.2	92.4	55.9	89.04	7.44
Sesquiterpene hydrocarbons							
78	*α*-Copaene	0.48	0.1	tr			
79	Caryophyllene	0.97	0.4	0.1			7.19
80	Humulene	0.07					0.59
81	*allo*-Aromadendrene	0.19					
82	Selina-4,11-diene	0.13					0.97
83	Germacrene D	1.02	2.8	1.8			0.99
84	Bicyclogermacrene	2.06	0.5	0.8			
85	*γ*-Cadinene	0.16					0.48
86	*α*-Cedrene						0.22
87	*β*-Farnesene		0.2	0.1			
88	*β*-Chamigrene		0.1				
89	Valencene						1.20
90	Ledene					0.12	2.17
91	Aciphyllene						1.34
92	Bulnesene						0.84
93	*β*-Bisabolene		0.2				
94	*δ*-Cadinene		0.1	tr			0.42
95	*β*-Elemene					0.71	0.84
Total sesquiterpene hydrocarbons		5.08	4.4	2.8	0	0.83	17.25
Oxygenated sesquiterpenes							
96	Spathulenol	1.10	0.7	0.2	0.2	0.17	1.97
97	Caryophyllene oxide	0.60	0.2	0.1	0.1		5.82
98	dehydro-Sesquicineol						0.9
99	Davana ether			0.1			
100	Davanone			1.3			
101	Viridiflorol		0.4				
102	Ledol		0.1				
103	Cedrol						2.27
104	Eremoligenol						0.69
105	Germacrene-D-1,10-epoxide		0.3				
106	*α*-Cadinol		0.1		0.1		
107	Germacra-4(15),5,10(14)-trien-1α-ol		0.1	0.1	0		
108	*α*-Bisabolol						0.42
109	4-Cuprenen-1-ol			tr			
110	Aciphilyc acid						0.91
Total oxygenated sesquiterpenes		1.70	1.9	1.8	0.4	0.17	12.98
Non-oxygenated hydrocarbons							
111	1-phenyl-2,4-Pentadiyne			0.1			
Total non-oxygenated hydrocarbons		0	0	0.1	0	0	0
Oxygenated hydrocarbons							
112	4-phenyl-2-Butanol	3.58			3.4		
113	4-phenyl-2-Butanone	34.95			33.1		
114	α-methyl-Benzenepropanol acetate	3.43					
115	(*2E*)-Hexenal			0.1			
116	Benzaldehyde				0.1		
117	1-Octen-3-ol		0.1				
118	(*2E*)-Dodecenal		0.2				
119	Phloacetophenone 2,4-dimethylether		0.3				
Total oxygenated hydrocarbons		41.96	0.6	0.1	36.6	0	0
Total monoterpenes		51.26	87.3	94.9	58.5	93.37	58.26
Total sesquiterpenes		6.78	6.3	4.6	0.4	1.00	30.23
Total hydrocarbons		41.96	0.6	0.2	36.6	0	0

* tr, trace amounts (less than 0.10%).
